# Healthy Physical and Mental Development of Children

**Published:** 1920-10-02

**Authors:** 


### Healthy Physical and Mental Development of Children.

The Draft, dated March 11, 1920, of the Consolidated Regulations relating to the Special Services of Elementary Education (other than Nursery Schools) for Promoting the Healthy Physical and Mental Development of Children having been published for the required period, has now been confirmed by the Board of Education without any important amendment in the Regulations themselves. Attention is drawn to paragraph 3 (*c*) of the Explanatory Memorandum, expressing the Board's opinion that any scheme of a Local Education Authority for open-air schools on a large scale, whether in the form of camp schools or otherwise, for ailing children selected by the school medical officer on account of their defective health, should be recognised under the Special Schools Regulations when the fundamental object of the scheme is remedial. The Draft now becomes "The Board of Education (Healthy Physical and Mental Development of Children Elementary Education) Regulations, 1920." Grant Regulations, No. 19, and copies can be purchased through any bookseller (price 3d.), or directly from H.M. Stationery Office, Imperial House, Kingsway, London, W.C. 2 (price by post 4d.).

